# Global Burden of Alzheimer's disease and other dementias in adults aged 65 years and older, 1991–2021: population-based study

**DOI:** 10.3389/fpubh.2025.1585711

**Published:** 2025-07-01

**Authors:** Zhu Xiaopeng, Yu Jing, Lai Xia, Wang Xingsheng, Deng Juan, Long Yan, Li Baoshan

**Affiliations:** Department of Geriatrics, Chongqing University Central Hospital/Chongqing Emergency Medical Center, Chongqing, China

**Keywords:** Alzheimer's disease and other dementias, 65 years and older, Global Burden, GBD, old age

## Abstract

**Objectives:**

This study aims to analyze the prevalence, impact, and disparities of Alzheimer's disease and other dementias (ADRD) among adults aged 65 years and older worldwide, across different regions and countries, spanning the years 1991–2021.

**Methods:**

GBD 2021 obtained data on dementia from vital registration systems, published scientific literature and surveys, and data from health-service encounters on deaths, excess mortality, prevalence, and incidence from 1991 to 2021, through systematic review and additional data-seeking efforts. Individuals aged ≥65 years from 21 regions and 204 countries and territories (Global Burden of Disease and Risk Factors Study 2021) from 1991 to 2021 were analyzed. Primary outcomes were ADRD related to aged ≥65 years age-standardized prevalence, deaths, disability-adjusted life years (DALYs), and average annual percentage change (AAPC), and the fractions of these metrics that were attributable to three risk factors (high BMI, high fasting glucose, and smoking) that met GBD.

**Results:**

The global mortality of ADRD among adults aged ≥65 years increased by 115%, from 6.5 (95% UI 1.5–18) per 100,000 population in 1991 to 14 (95% UI 3.5–37) per 100,000 population in 2021, with an Average Annual Percentage Change (AAPC) of 1.10% (95%CI 0.45%−1.76%). The prevalence of ADRD in adults aged ≥65 years increased by 160% between 1991 and 2021, from 18.7 (95%UI 14.9–23.2) million to 49 (95%UI 38.6–61.2) million. The aged ≥65 years age-standardized prevalence of ADRD in this age group increased from 11,977 (95%UI 9,438–14,935) per 100,000 population in 1991 to 12,124 (95%UI 9,489–15,204) per 100,000 population in 2021. The aged ≥65 years the number of prevalent persons was more significant among females than among males (males: from 6.2 (95%UI 4.8–7.8) million in 1991 to 17.2 (95%UI 13.4–21.6) million in 2021; women: from 12.5 (95%UI 10.0–15.4) million in 1991 to 31.7 (95%UI 25.1–39.6) million in 2021). In 2021, ADRD in adults aged ≥65 years caused 8.02 (95%UI 1.34–22.19) million deaths and 25.38 (95%UI 23.18–71.20) million DALYs attributable to dementia, and high BMI, high fasting glucose, and smoking remained modifiable risk factors in all risk factors.

**Conclusions:**

From 1991 to 2021, the worldwide prevalence of Alzheimer's disease and other dementias among adults aged 65 and above has increased by 1.6 times, largely driven by the expanding older adults. This escalating trend poses significant challenges to the global healthcare system in terms of both mortality rates and disability-adjusted life years. We recommend that standardized screening programmes be promoted globally, especially in high-risk areas and among high-risk populations, for early detection and intervention of the disease. Secondly, we recommend strengthening the management of risk factor elements.

## Introduction

Alzheimer's disease and other dementias (ADRD) pose a significant global health challenge, particularly among adults aged 65 years and older (1, 2). Over the past three decades, the increased prevalence of ADRD, as well as mortality and DALYs attributable to people with ADRD, have increased with age (3), reflecting demographic shifts such as increased life expectancy and aging populations. Despite advancements in medical research, the exact causes of ADRD remain unclear, complicating efforts to develop effective treatments and preventive measures (4, 5). The increasing prevalence of dementia, particularly Alzheimer's disease, is estimated that by 2050, there will be 152 million people living with ADRD (6), underscores the urgent need for effective strategies in the prevention, diagnosis, and management (7). The profound social and economic impacts affect individuals, their families, and healthcare systems worldwide (8). As populations age, the demand for dementia care is projected to escalate, necessitating a robust understanding of epidemiological patterns and risk factors associated with these conditions (9, 10).

This study is based on population data from different regions, although many previous studies have utilized publicly available data from the Global Burden of Disease (GBD) studies analyzing incidence, mortality, and disability-adjusted life years (DALYs) for ADRD, as well as for other neurological conditions (2, 11). Our study is innovative in that we focused on the incidence, mortality, and disability-adjusted life years (DALYs) associated with ADRD among people aged 65 years and older globally during the period from 1991 to 2021. In the analysis, we also took into account the level of social development, age and gender of the older population. This is not only a further update of the existing data, but more critically, we focus on the older adults as a major morbidity group and delve deeper into the main populations affected by ADRD. The primary objective of this study is to offer guidance to policymakers, healthcare professionals, and scholars to encourage the implementation of targeted interventions and support systems to address the escalating burden of dementia on a global scale.

## Methods

### Study population and data collection

The Study of Disease Burden on a Global Scale is a joint scientific endeavor that enables the comparison and reproduction of the impacts of various health conditions across age groups, genders, and geographic regions at specific time intervals. The study analyzed ADRD in 21 regions and 204 countries/territories between 1991 and 2021 using online global health data updated on May 16, 2024 (http://ghdx.healthdata.org/gbd-results-tool). The study population was 65 and older, including those diagnosed with dementia before age 65. The study extracted disease information, including prevalence, mortality, and DALYs, for men and women in different age groups over 65, considering different sociodemographic indices. The study defined ADRD, using a physician's diagnosis as the disease recognition criterion, and calculated the corresponding prevalence, mortality, and DALYs (Prevalence, mortality and DALYs, available through the GBD website). Age-standardized rates in GBD are estimated using the GBD world population age standard. Briefly, A standard population structure was generated by calculating the non-weighted average of all countries over all years for adults aged 65 and above worldwide. Years of life lost were calculated by multiplying the predicted deaths by a predetermined life expectancy criterion to arrive at a total number of disability-adjusted life years (DALYs). Utilizing data from this collective effort, our research did not entail direct engagement with the study participants. Patient involvement needed to be present in formulating the research inquiry or defining the outcome metrics, and they needed to be more engaged in designing or executing the study. This study used data from publicly available databases and did not require ethical approval.

### Statistical analysis

We performed descriptive analyses comparing age-standardized prevalence, mortality, and years of disability (DALYS) for Alzheimer's disease and other dementias in adults ≥65 years of age across age groups, sexes, regions, and countries globally. Based on data from the Global Burden of Disease Study, we assessed risk factors for mortality and DAYLS. We used connected-point regression analyses fitted to logit models to describe trends over specific periods, and different interval-specific disease variant characteristics were evaluated. We estimated the prevalence of ADRD in adults ≥65 years of age calculated average annual percent change (AAPC) and annual percent change (APC) using the pandas and statsmodels libraries in Python to assess whether trends in disease incidence were statistically significant across intervals. In the analysis, we considered the corresponding rate upward or downward if the annual percentage change estimate and the 95% CI deviated meaningfully from zero and considered statistically significant if the *p*-value was <0.05.

## Results

### Global trends

The Global mortality of ADRD among adults aged 65 years and older increased by 115%, from 6.5 (95% UI 1.5–18) per 100,000 population in 1991 to 14 (95% UI 3.5–37) per 100,000 population in 2021, with an Average Annual Percentage Change (AAPC) of 1.10%(95%CI 0.45 to 1.76%; Table 1). Between 1991 and 2021, the prevalence of ADRD among adults aged 65 years and older increased by 160 percent, from 18.7 (95%UI 14.9–23.2) million to 49 (95%UI 38.6–61.2) million. The age-standardized prevalence of ADRD in this age group increased from 11,977 (95%UI 9,438–14,935) per 100,000 population in 1991 to 12,124 (95%UI 9,489–15,204) per 100,000 population in 2021, AAPC of 0.05% (95%CI −0.26% to 0.35%; Supplementary Table 1). The burden of disease (DALYs) for ADRD among persons aged 65 years and older has been increasing over the past 30 years. Age-standardized DALYs rose from 779 (95%UI 109–2,281) per 100,000 population in 1991 to 1,009 (95%UI 76–2,978) per 100,000 population in 2021, a 30% increase Average Annual Percentage Change (AAPC 0.47%, 95%CI 0.27%−0.67%; Supplementary Table 2).

**Table 1:** Age standardized death and AAPC of Alzheimer's disease and other dementias in people aged ≥65 years at global and regional level, 1991–2021.

	**Death (95% UI) No of people with Alzheimer's disease and other dementias in 1991 (000s)**	**Age standardized rate in 1991 (per 100 000)**	**No of people with Alzheimer's disease and other dementias in 2021 (000s)**	**Age standardized rate in 2021 (per 100 000)**	**AAPC (95% CI)**	***P*-value**
Global	651 (157–1,791)	860 (204–2,311)	1,404 (352–3,692)	880 (216–2,275)	1.10 (0.45–1.76)	< 0.01
**Sex**						
Female	191 (112–1,244,559.2)	944 (225–2,500)	952 (244–2,433)	982 (24–2,493)	1.25 (0.65–1.86)	< 0.01
Male	459 (445–544)	698 (159–1,942)	452 (108–1,259)	701 (165–1,901)	0.98 (0.32–1.65)	< 0.01
**Age group (years)**						
65–69	38 (8–114)	29 (7–89)	64 (15–189)	31 (7–90)	1.62 (0.90–2.34)	< 0.01
70–74	55 (13–1,590)	62 (15–177)	99 (25–282)	63 (16–179)	1.53 (0.79–2.28)	< 0.01
75–79	89 (20–262)	138 (32–410)	145 (35–417)	139 (33–402)	1.33 (0.60–2.07)	< 0.01
80–84	166 (40–454)	418 (100–1,162)	293 (74–777)	422 (104–1,145)	1.04 (0.33–1.76)	< 0.01
85–89	163 (40–441)	935 (224–2,552)	349 (88–912)	949 (235–2,547)	0.85 (0.25–1.45)	< 0.01
90–94	97 (23–251)	1,867 (445–4,980)	283 (72–699)	1,911 (473–4,878)	0.68 (0.17–1.20)	< 0.01
≥95	41 (10–107)	3,409 (801–9,058)	168 (43–414)	3,505 (859–8,899)	0.65 (0.22–1.08)	< 0.01
**SDI level**						
High	283 (70–755)	1,087 (273–2,779)	706 (183–1,774)	1,043 (272–2,545)	1.39 (0.11–2.69)	0.03
High-middle	168 (40–465)	988 (237–2,654)	441 (109–1,176)	1,003 (250–2,592)	1.41 (0.03–2.79)	0.04
Middle	19 (4–56)	723 (159–2,020)	54 (13–154)	839 (195–2,266)	0.90 (−0.57 to 2.39)	0.23
Low-middle	55 (13–159)	656 (151–1,813)	177 (42–494)	744 (177–1,969)	0.91 (−0.56 to 2.39)	0.23
Low	19 (4–56)	723 (159–2,020)	54 (13–154)	839 (195–2,266)	0.90 (−0.57 to 2.39)	0.11

AAPC, average annual percentage change; CI, confidence interval; SDI, sociodemographic index; UI, uncertainty interval.

### Global trends by sex

From 1991 to 2021, the age-standardized prevalence of ADRD increased globally among both men and women in the 65-and-over age group, but more women than men [males: from 6.2 (95%UI 4.8–7.8) million in 1991 to 17.2 (95%UI 13.4–21.6) million in 2021; women: from 12.5 (95%UI 10.0–15.4) million in 1991 to 31.7 (95%UI 25.1–39.6) million in 2021]). The prevalence of ADRD increased at a faster rate in men than in women AAPC 0.06% (95%CI −0.25% to 0.37%) vs. 0.03% (95%CI −0.24% to 0.31%). At the same time, age-standardized death rates for ADRD for men and women aged 65 and older also trended upward, with men increasing at a slightly slower rate than women AAPC 0.98% (95%CI 0.32–1.65%) vs. 1.25% (95%CI 0.65%−1.86%). Over the same period, age-standardized DALYs due to ADRD increased for both those aged 65 years and older, with a higher rate of increase for women than for men [men: from 3.2 (95%UI 0.8–8.6) million to 10.2 (95%UI 1.7–27.8) million; women: from 4.8 (95%UI 0.5–13.7) million to 15.2 (95%UI 0.5–43.6) million]. This gender difference persists across socio-demographics, with women having a higher burden of disease than men, especially in countries with high-medium Sociodemographic indices (Supplementary Figures 1, 2). To ensure the accuracy of the AAPC, we looked at trends in the number of people with the disease by plotting trends and calculating the APC over time, and the increase in mortality among people aged ≥65 years women was consistently higher than man (Supplementary Figures 3, 4).

### Global trends by age subgroup

Between 1991 and 2021 on a global scale, the occurrence of ADRD in individuals aged 65 and above has increased significantly in every age bracket (65–69 years: from 230,000 to 540,000; 70–74 years: from 290,000 to 740,000; 75–79 years: from 410,000 to 930,000; 80–84 years: from 460,000 to 1.14 million; 85–89: from 310,000 to 920,000), with a fourfold rise observed among those aged 90 and above (90–94 years: from 120,000 to 470,000; ≥95 years: 36,000 to 180,000). It is noteworthy that in all age groups, the increase in prevalence for females consistently outpaced that for males (Supplementary Figures 5–7). The standardized prevalence of ADRD experienced a slow increase across all age categories except for individuals aged 65–69 years (AAPC 0.05%, 95%CI −0.26% to 0.35%) from 1991 to 2021 (Supplementary Table 1). Globally, standardized mortality rates for ADRD rose across all age groups within the older population, particularly among those below the age of 84. By 2021, mortality rates for ADRD among individuals aged 65 and above escalated with age, increasing from 31 per 100,000 individuals in the 65–69 age bracket to 3,505 per 100,000 individuals in the ≥95-year age group, marking a 100-fold surge (Table 1). Standardized DALYs for ADRD displayed varying growth rates across different age categories. The most notable upsurges in AAPC (95%CI) were observed in age groups under 84, including 65–69 years 0.53% (0.10%−0.97%), 70–74 years 0.54% (0.06%−1.02%), 75–79 years 0.58% (0.04%−1.12%), and 80–84 years 0.56% (−0.05 to 1.17%). In 2021, DALYs attributable to ADRD increased with age, escalating from 137 per 100,000 population in the 65–69 age group to 3,012 per 100,000 population in those aged ≥95 years, reflecting a 20-fold rise (Supplementary Table 2).

### Global trends by sociodemographic index

Between 1991 and 2021, age-standardized mortality rates for ADRD in the 65-and-over age cohort show an increasing trend in all subgroups of the sociodemographic index, especially in countries with a medium sociodemographic index (AAPC 1.41%, 95%CI 0.03%−2.79%). Regardless of sociodemographic level, the increase in mortality in the 65-and-over age group was consistently higher than in the general population (Supplementary Figures 3, 4). As of 2021, among the older age groups, countries with high sociodemographic indices have the highest mortality rates for ADRD (1,043 per 100,000 population; Table 1). At the same time, the age-standardized prevalence of ADRD in 2021 tended to increase in countries with a high sociodemographic index (AAPC 0.30%, 95%CI 0.26%−0.34%). In contrast, it tended to decrease in countries with a low sociodemographic index (AAPC −0.15%, 95%CI −0.163 to −0.13). Countries with a high sociodemographic index have the highest prevalence in 2021 (14,425 per 100,000 population), more than double that of countries with a low sociodemographic index (9,612 per 100,000 population). In contrast, the fastest prevalence growth between 2010 and 2021 is in countries with a high-middle sociodemographic index (Supplementary Figures 8, 9).

Age-standardized DALYs for ADRD increased significantly in all sociodemographic subgroups except those with a high sociodemographic index (AAPC −0.01%, 95%CI −0.03% to 0.01%; Supplementary Table 1). The trend toward increased AAPC for AD and other dementia DALYs was more pronounced with decreasing values of the sociodemographic index (Supplementary Table 2). In 2021, DALYs were lowest in countries with a low sociodemographic index (634 per 100,000 population) and highest in countries with a moderately high sociodemographic index (1,322 per 100,000 population; Supplementary Table 2) but low sociodemographic index age-standardized DALYs with ADRD in the older adults population had the fastest increase in AAPC.

### Regional and national trends

From 1991 to 2021, there were significant differences in the age-standardized AAPC in ADRD among people aged 65 years and older across 21 regions. During 1991–2000, the fastest growth was in the high-income Asia-Pacific region (4.64%), while during 2011–2021, the most rapid growth was in East Asia (4.48%). In relative terms, the slowest growth was in the Nordic region (−1.40%) in 1991–2000 and Western Europe (−1.24%) in 2001–2010, respectively (Supplementary Figure 10).

**Figure 1:**
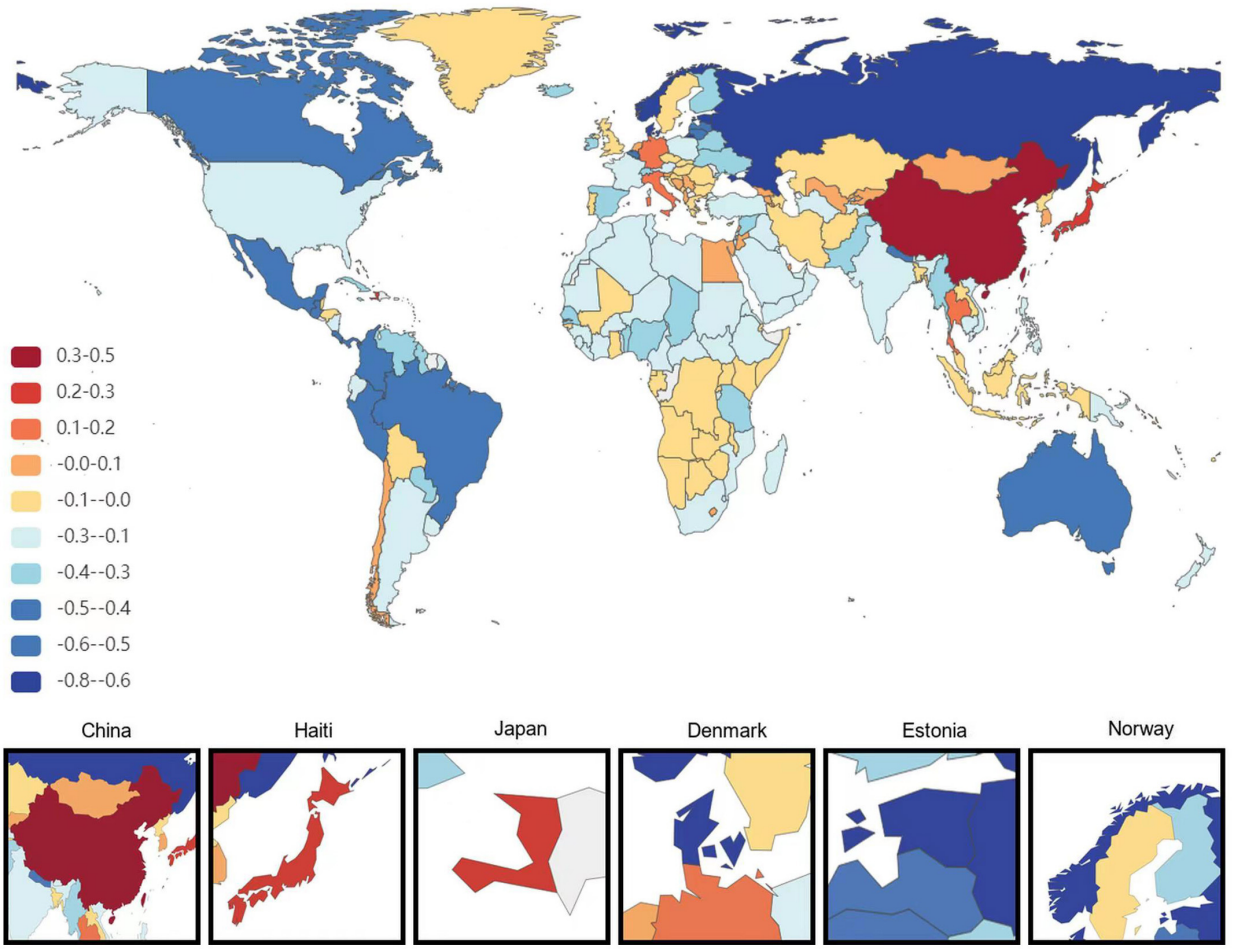
Map showing average annual percentage change in global prevalence of Alzheimer's disease and other dementias among people aged ≥65 years, 1991–2021.

**Figure 2:**
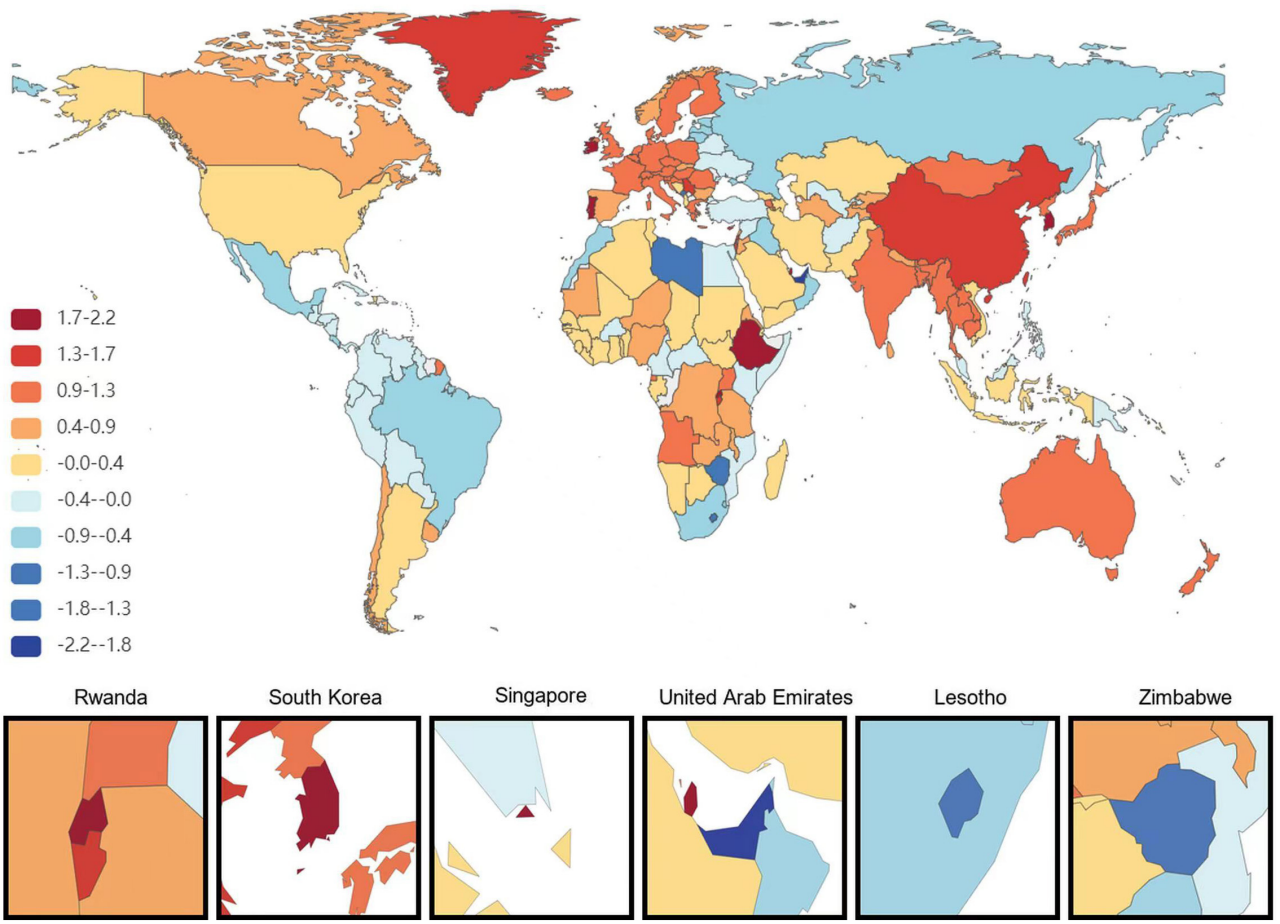
Map showing average annual percentage change in global DALYs of Alzheimer's disease and other dementias among people aged ≥65 years, 1991–2021.

**Figure 3:**
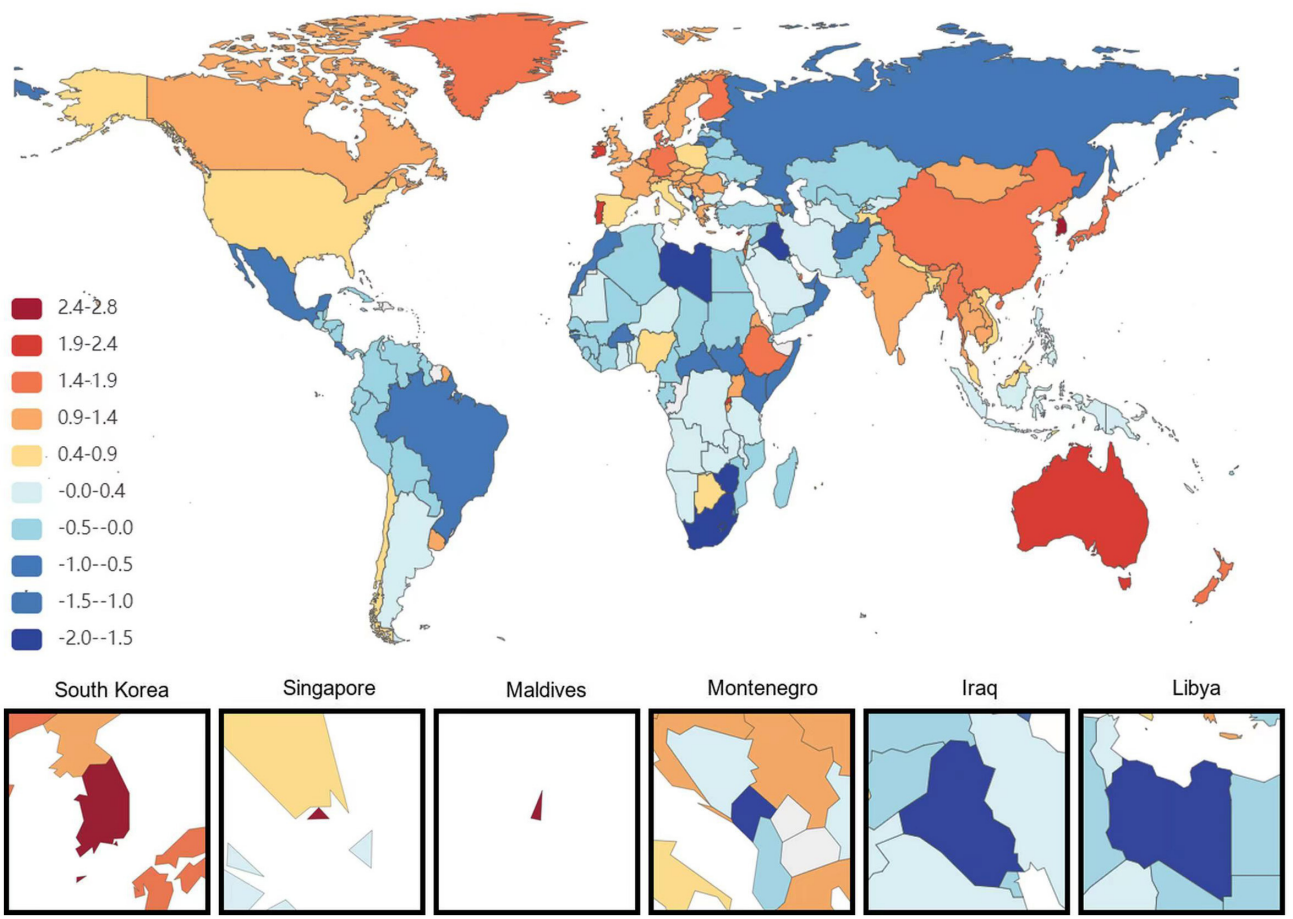
Map showing average annual percentage change in global mortality of Alzheimer's disease and other dementias among people aged ≥65 years, 1991–2021.

At the national level, China had the most significant increase in the age-standardized prevalence of ADRD among people aged 65 years and older from 1991 to 2021, with an average annual increase of 0.84%. This is followed by Haiti (0.75% AAPC) and Japan (0.75%AAPC), respectively, and the most minor dcreases are in Estonia and Denmark, with AAPC of −0.73% and −0.67%, respectively. During the same period, the countries with the most significant increases in age-standardized DALYs for ADRD in people aged 65 years and older were South Korea and Sweden (AAPC 2.18 and 2.16%), followed by Singapore (1.97%). The minor increases were in the United Arab Emirates and Lesotho, with AAPC of −2.21 and −1.36%, respectively. The country with the most significant increase in age-standardized mortality rates for ADRD was South Korea (AAPC of 2.84%), followed by Singapore (2.67%). The most minor average annual increases were in Montenegro (AAPC −1.95%) and Iraq (AAPC −1.88%; Figures 1–3).

### Risk factors

We selected the top three risk factors based on a comprehensive analysis of statistical parameters, particularly *p*-values, odds ratios (ORs), and average annual percentage change (AAPC). A detailed analysis of global data from 1991 to 2021 revealed that the three have a great influence risk factors associated with Alzheimer's disease (AD) and other dementia symptoms (DALYs) among people aged ≥65 years, included high fasting glucose levels, high BMI, and tobacco (Table 2). In 2021, these factors accounted for 1,638, 833, and 291 per 100,000 population with DALYs, respectively. Between 1991 and 2021, these factors corresponded to AAPC (95%CI) of 1.21% (1.08%−1.33%), 1.31% (1.19%−1.42%), and −0.97% (−1.50% to −0.43%), respectively. The three main risk factors for deaths associated with AD and other dementia symptoms among people aged ≥65 years globally during this period included high fasting glucose levels, high BIM, and tobacco (Supplemental Table 3). In 2021, these factors accounted for 134 deaths per 100,000 people, 70 deaths per 100,000 people, and 22 deaths per 100,000 people, respectively. Between 1991 and 2021, these factors corresponded to an AAPC (95%CI) of 1.21% (1.08%−1.33%), 1.32% (1.20%−1.43%), and −0.99% (−1.53% to −0.45%), respectively.

**Table 2:** Main risk factors for age standardized Alzheimer's disease and other dementias related DALYs, and AAPC, among people aged ≥65 years, 1991–2021.

**Risk factors by SDI**	**Age standardized DALYs rate (95% UI) 1991**	**Age standardized DALYs rate (95% UI) 2021**	**AAPC (95% CI)**	***P*-value**
**High fasting plasma glucose**				
Global	1,159 (66–3,268)	1,638 (92–4,596)	1.21 (1.08–1.33)	< 0.01
High SDI	1,283 (75–3,571)	1,867 (107–5,252)	1.43 (1.29–1.58)	< 0.01
High-middle SDI	1,135 (63–3,243)	1,575 (90–4,422)	1.07 (0.96–1.18)	< 0.01
Low SDI	784 (41–2,224)	1,098 (55–3,249)	0.99 (0.78–1.20)	< 0.01
Low-middle SDI	825 (45–2,284)	1,290 (69–3,669)	1.32 (1.15–1.49)	< 0.01
Middle SDI	1,090 (58–3,138)	1,456 (80–4,127)	0.88 (0.75–1.01)	< 0.01
**High body-mass index**				
Global	624 (−48 to 2,539)	833 (−124 to 3,182)	1.31 (1.19–1.42)	< 0.001
High SDI	862 (−100 to 3,373)	997 (−163 to 3,669)	0.86 (0.67–1.04)	< 0.001
High-middle SDI	705 (−75 to 2,943)	1,020 (−173 to 3,791)	1.30 (1.17–1.43)	< 0.001
Low SDI	49 (−30 to 382)	209 (−5 to 1,064)	4.99 (4.39–5.59)	< 0.001
Low-middle SDI	153 (−0.9 to 764)	440 (−49 to 1,712)	3.23 (3.01–3.45)	< 0.001
Middle SDI	215 (−1.4 to 1,066)	584 (−69 to 2,334)	3.812 (3.56–4.08)	< 0.001
**Tobacco**				
Global	399 (153–922)	291 (114–668)	−0.97 (−1.50 to −0.43)	< 0.001
High SDI	465 (182–1,072)	292 (114–661)	−1.37 (−1.983 to −0.74)	< 0.001
High-middle SDI	346 (128–801)	324 (122–753)	−0.30 (−0.94 to 0.34)	0.35
Low SDI	187.1 (72.2–450.6)	150.9 (54–372)	−0.92 (−1.16 to −0.67)	< 0.001
Low-middle SDI	298 (116–714)	233 (88–558)	−1.00 (−1.30 to −0.70)	< 0.001
Middle SDI	382 (146–887)	301 (116–712)	−0.99 (−1.48 to −0.50)	< 0.001

AAPC, average annual percentage change; CI, confidence interval; SDI, sociodemographic index; UI, uncertainty interval.

## Discussion

Over the past 30 years, despite improvements in global health care for the older population aged 65 years and older, age-standardized disability-adjusted life-years (DALYs) rates and mortality associated with ADRD have increased and remain one of the heaviest economic burdens of disease, in line with previous reports (12). Despite the relatively slow increase in prevalence, these diseases remain a significant factor in the life expectancy of older adults. Our study extends the understanding of the growing global burden by focusing on trends in ADRD in older adults aged 65 years and older. This is important for improving older people's health and directs health practice and future research (12).

### Age and sex differences in the burden of ADRD in older adults

Our study found that the prevalence of ADRD is growing slowly in 2021. This may be attributed to advances in early diagnosis and intervention, treatment, and care, as well as increased health awareness among the population (13). However, as ADRD are mostly progressive and irreversible diseases, the growing aging population and increasing diagnosis rates, combined with the current lack of effective treatments and the impact of various co-morbidities and psychosocial factors (14), have led to an increasing risk of death and disability associated with these diseases, which is in line with the findings of previous studies (15). Segmented analyses revealed changes in the prevalence of ADRD in each age group itself. In age-standardized prevalence comparisons, the interference of differences in the age structure of the population can be eliminated by calculating age-standardized prevalence rates at different time points. We found that the risk of death and disability from ADRD was significantly increased in every age subgroup among those ≥65 years of age. The age-standardized rate was considerably higher, and the increase in age-standardized rate further emphasizes the public health challenges posed by aging societies and the urgent need for comprehensive measures in healthcare, care, and social support to mitigate the burden and impact of these diseases burden and impact (13, 16). Despite slight declines in some age groups, population aging and growth will lead to further increases in the prevalence of dementia in the absence of major scientific breakthroughs.

In people aged 65 years and older, we observed a higher overall prevalence of ADRD in women compared to men (17). This may be because women generally live longer than men on average, the decrease in estrogen levels in women after menopause, and specific genetic effects such as APOE ε4 (18–20). Cerebral blood flow and metabolic rates in women differ from those of men, and women are more susceptible to autoimmune diseases that may indirectly affect the immune system and increase the risk of dementia (21). We found that the prevalence and disability rates of AAPC were lower in women than in men, which may be due to women's greater emphasis on early screening, regular medical checkups, and preventive treatments, more active lifestyles, health behaviors, and preventive measures, as well as socio-medical advances.

### Regional and national differences in the burden of ADRD in older people

The average annual prevalence of ADRD is increasing slowly in people aged 65 years and older, with inequalities between countries (22). In Asian countries such as China, Korea, and Japan, the prevalence is increasing faster, while European countries such as Denmark are experiencing negative growth. This phenomenon may be due to the decline in birth rate, longer average life expectancy, and the rapid increase in the proportion of older adults (22), as well as high sugar and fat diets and lack of physical activity due to modern dietary shifts in Asia, where smoking is more prevalent in men than in other regions (23, 24). It is also possible that advances in medical technology and diagnostic tools in the area have made early diagnosis of ADRD more prevalent, leading to an increase in prevalence, which is consistent with previous findings (25).

Among people aged 65 years and older, the annual average percentage change (AAPC) in disability-adjusted life years (DALYs) and mortality for ADRD is higher than the prevalence rate. This may be because the disease progressively worsens, leading to loss of function and a severe decline in the quality of life of the patient. Despite the increased level of awareness and diagnosis of dementia, effective treatments are still limited (26, 27). In many Asian countries, where aging is occurring at a faster rate, health systems may not be fully prepared to deal with the needs of a large number of people with dementia. In contrast, the situation is different in developed countries such as Europe (28).

We analyze the relationship between socio-demographic indices (SDI) and the burden of disease, taking into account differences in healthcare infrastructure. Low SDI areas may face challenges in disease prevention, diagnosis, and treatment due to limited healthcare resources. We suggest that for low SDI areas, priority should be given to improving basic healthcare services, such as increasing access to primary care physicians and diagnostic facilities, and that policymakers in high SDI areas focus on the development of long-term care policies to support growing older adults populations; while low SDI areas should invest more in public health education and disease surveillance systems.

In addition to SDI factors, such as characteristics of the health care system, cultural factors, or differences in policy implementation in different regions, may also influence the prevalence of ADRD in older people.

### Risk factors for the burden of ADRD in older adults

In people aged ≥65 years, high fasting glucose levels and high BMI were identified as significant factors associated with ADRD leading to DALYS and deaths, which is the same as previous findings (7, 29). It may be that high fasting blood glucose affects glucose utilization in the brain, leading to impaired energy metabolism. Hyperglycemia may inhibit IDE activity and reduce Aβ degradation, while hyperglycemia is associated with abnormal phosphorylation of tau proteins (30). High BMI is associated with cerebrovascular diseases such as hypertension and atherosclerosis (31), which increase the risk of insufficient cerebral blood supply, which in turn affects cognitive function. On the other hand, high BMI is associated with systemic inflammation and insulin resistance, and chronic inflammation and oxidative stress lead to neuronal damage and Alzheimer's disease-related brain lesions (32). Also, BMI is unstable, and improving cognition becomes more challenging for older adults due to metabolic disorders and other factors associated with aging (33). Global reductions in smoking exposure and negative increases in Deaths and DAYLS due to ADRD suggest that success in reducing smoking through regulatory policies may point the way to a more significant role for public policy in other risks (34).

Policy Recommendations: 1. Community-based Screening and Education: Set up regular diabetes screening camps in high-risk communities, offering free blood glucose and HbA1c tests. Conduct educational workshops on healthy living and early detection. 2. Multi-media Awareness Campaigns: Launch comprehensive anti-smoking campaigns on various media, use influencers and celebrities. 3. Policy-level Interventions: Increase tobacco taxes and implement strict smoke-free laws in public and indoor areas. 4. Healthcare Provider Training: Train healthcare providers on cognitive health assessment and promotion, incorporate it into geriatric care guidelines.

### Limitations of this study

This study has several limitations. First, the data were extrapolated using existing GBD data models, and caution is needed to interpret these results. For example, in some areas with limited medical resources, the surveillance and reporting of diseases may be incomplete, which could lead to an underestimation of the actual disease burden in the relevant data. Second, there are differences in health information systems and reporting mechanisms across countries and regions (22), which may lead to incomplete and biased data and affect the accuracy of the results. Third, there needs to be a time lag in the burden of disease data. Fourth, the choice of models and parameters may affect the results (35). Finally, Diagnostic Criteria: the study uses physician diagnosis as the criterion for ADRD, there are multiple types of dementia in the older adults, and diagnosis is challenged by differences in healthcare systems, policies, and practices across countries (36). While risk factors are identified, the study design does not establish causality.

## Conclusions

Mortality and disability-adjusted life years (DALYs) in older patients aged 65 years and above with ADRD showed a significant increase from 1991 to 2021, albeit at a slower rate of prevalence growth. The disease is more prevalent in women than men, and mortality and DALYs tend to rise with age among individuals with ADRD. The annual prevalence rate is rising more rapidly in the middle and high-economic regions of Asia, including countries like China, South Korea, and Japan. Managing high fasting glucose levels and body mass index (BMI) poses a major challenge for older adults with ADRD. The prevention and treatment of dementia are expected to present a growing challenge to healthcare systems worldwide.

## Data Availability

Publicly available datasets were analyzed in this study. This data can be found here: https://ghdx.healthdata.org/gbd-2021 —GBD 2021.
