# Supplementary material for: Global Burden of Alzheimer's disease and other dementias in adults aged 65 years and older, 1991–2021: population-based study
Source: Front Public Health. 2025 Jul 1;13:1585711. doi: 10.3389/fpubh.2025.1585711 (PMC12261920; doi:10.3389/fpubh.2025.1585711)
Supplement: Supplementary file 1 [file Data_Sheet_1.docx]

**Supplementary Table 1** Age standardised prevalence and AAPC of Alzheimer’s disease and other dementias in people aged ≥65 years at global and regional level, 1991-2021

**Supplementary Table 2** Age standardised DAYLS and AAPC of Alzheimer’s disease and other dementias in people aged ≥65 years at global and regional level, 1991-2021

**Supplementary Table 3** Main risk factors for Age standardised Alzheimer’s disease and other dementias related death, and AAPC, among people aged ≥65 years , 1991-2021

**Supplementary Figure 1** Gender differences in the prevalence of Alzheimer's disease and other dementias

**Supplementary Figure 2** Regional differences in the prevalence of Alzheimer's disease and other dementias

**Supplementary Figure 3** Comparison of Alzheimer's disease and other dementia prevalence, mortality, and DALYS by gender in the total population

**Supplementary Figure 4** Prevalence of Alzheimer's Disease and Other Dementias, Mortality, and DALYS Across Sexes in People ≥65 Years of Age

**Supplementary Figure 5** Prevalence of Alzheimer's disease and other dementias among people aged ≥65 years . (both = Male + female)

**Supplementary Figure 6**  Deaths of Alzheimer's disease and other dementias among people aged ≥65 years . (both = Male + female)

**Supplementary Figure 7** DAYLS of Alzheimer's disease and other dementias among people aged ≥65 years . (both = Male + female)

**Supplementary Figure 8**  Alzheimer's disease and other dementias in Persons ≥65 Years of Age by Sociodemographic Indicator average annual percentage changes

**Supplementary Figure 9**  Prevalence, Mortality and Dalys of Alzheimer's Disease and Other Dementias in Persons ≥65 Years of Age by Sociodemographic Indicator

**Supplementary Figure 10** Prevalence, mortality, and DYLS of Alzheimer's disease and other dementias among people aged ≥65 years in different regions at three time periods

**Supplementary Table 1 Age standardised prevalence and AAPC of** **Alzheimer’s disease and other dementias in people aged ≥65 years at global and regional level, 1991-2021**

|  | **Prevalence (95% UI)**  No of people with  Alzheimer’s disease and other  dementias in 1991 (00 000s) | Age standardised rate in 1991  (per 100 000) | No of people with  Alzheimer’s diseaseand other  dementias in 2021 (00 000s) | Age standardised rate in 2021  (per 100 000) | AAPC (95% CI)) |
| --- | --- | --- | --- | --- | --- |
| Global | 187(149 to 232) | 11977(9438 to 14935) | 490(386 to 612) | 12124(9489 to 15204) | 0.05(-0.26 to 0.35) |
| Sex: |  |  |  |  |  |
| female | 125(100 to 154) | 13037(10292 to 16221) | 317(251 to 396) | 13292(10428 to 16621) | 0.03(-0.24 to 0.31) |
| Male | 62(48 to 78) | 10151(7945 to 12738) | 172(134 to 216) | 10124(7850 to 12778) | 0.06(-0.25 to 0.37) |
| Age group  (years): |  |  |  |  |  |
| 65-69 | 23(18 to 29) | 1763(1377 to2225) | 54(42 to 68) | 1847(1437 to 2324) | 0.05(-0.16 to 0.27) |
| 70-74 | 29(23 to 37) | 3258(2550 to 4133) | 74(57 to 94) | 3372(2621 to 4296) | -0.002( -0.23 to0.23) |
| 75-79 | 41(33 to 51) | 6376(5078 to 7816) | 93(74 to 115) | 6621(5249 to 8147) | -0.02(-0.289 to 0.25) |
| 80-84 | 46(37 to 57) | 11642(9325 to 14459) | 114(91 to 142) | 12002(9553 to 14985) | -0.05(-0.34 to 0.25） |
| 85-89 | 31(25 to 38) | 18028(14238 to 22327) | 92(72 to 113) | 18369(14449 to 22868) | -0.05（-0.35 to 0.25） |
| 90-94 | 12(10 to 15) | 24073(18996 to 29960) | 47(37 to 59) | 24199(18970 to 30375) | -0.05(-0.34 to 0.25) |
| ≥95 | 3.6(2.8 to 4.4) | 30047(23392 to 37844) | 18(14 to 22) | 29934(23071 to 37904) | -0.04(-0.30 to 0.22) |
| SDI level: |  |  |  |  |  |
| High | 74(60 to 90) | 14425(11616 to 17649) | 157(125 to 194) | 13289(10522 to 16550) | -0.01(-0.03 to 0.01) |
| High-middle | 49(39 to 61) | 13084(10336 to 16283) | 130(102 to 163) | 14425(11273 to 18087) | 0.30(0.26 to 0.34) |
| Middle | 39(31to 50) | 12151(9489 to 15255) | 140(110 to 177) | 13342(10413 to 16736) | 0.19(0.14 to 0.23) |
| Low-middle | 19(15 to 24) | 10249(7987 to 12932) | 48(38 to 61) | 9954(7725 to 12575) | -0.10(-0.12 to -0.08) |
| Low | 6(5 to 8) | 9977(7765 to 12558) | 14(11 to 18) | 9612(7512 to 12077) | -0.15(-0.163 to -0.13) |

AAPC=average annual percentage change; CI=confidence interval; SDI=sociodemographic index; UI=uncertainty interval.

**Supplementary Table 2 Age standardised DAYLS and AAPC of Alzheimer’s disease and other dementias in people aged ≥65 years at global and regional level, 1991-2021**

|  | **DAYLS (95% UI)**  No of people with  Alzheimer’s disease and other  dementias in 1991(000s) | Age standardised rate  in 1991 (per 100 000) | No of people with  Alzheimer’s diseaseand other  dementias in 2021 (000s) | Age standardised rate  in 2021 (per 100 000) | AAPC (95% CI)) |
| --- | --- | --- | --- | --- | --- |
| Global | 8018(1340 to 22191) | 779(109 to 2281) | 25376(2318 to 71196) | 1009(76 to 2978) | 0.47（0.27 to 0.67） |
| Sex: |  |  |  |  |  |
| female | 4786（497 to 13738) | 761(75 to 2291) | 15182(514 to 43575) | 1028(42 to 3081) | 0.31（0.05 to 0.57) |
| Male | 3232（817 to 8572) | 807(169 to 2270) | 10194(1740 to 27772) | 971(132 to 2811) | 0.77（0.23 to1.33 ) |
| Age group  (years): |  |  |  |  |  |
| 65-69 | 1096（229 to 2974) | 109(23 to 299) | 2848(330 to 7798) | 137(16 to 379) | 0.53(0.10 to 0.97) |
| 70-74 | 1273（257 to 3448) | 184(37 to 499) | 3628(379 to 9987) | 232(25 to 646) | 0.54(0.06 to1.02) |
| 75-79 | 1542（265 to 4203) | 311(56 to 852) | 4052(401 to 11242) | 403(42 to 1129) | 0.58(0.04 to 1.12) |
| 80-84 | 1866（292 to 5115) | 633(106 to 1731) | 5514(526 to 15031) | 814(83 to 2237) | 0.56(-0.05 to 1.17) |
| 85-89 | 1385（188 to 3958) | 1059(159 to 3006) | 4929(400 to 14065) | 1364(123 to 3915) | 0.45(-0.16 to 1.07) |
| 90-94 | 633（75 to 1867) | 1594(219 to 4689) | 3031(187 to 8956) | 2066(155 to 6158) | 0.34(-0.26 to0.95) |
| ≥95 | 222（21 to 686) | 2309(268 to 7075) | 1372(61 to 4193) | 3012(164 to 9238) | 0.27(-0.34 to 0.88) |
| SDI level: |  |  |  |  |  |
| High | 3534（511 to 9785) | 1143(128 to 3343) | 9326(589 to 26108) | 1322(70 to 3845) | -0.01(-0.42 to 0.40) |
| High-middle | 2074（305 to 5861) | 955(96 to 2899) | 6525(555 to 18214) | 1237(63 to 3657) | 0.47(0.08 to 0.87) |
| Middle | 1579(346 to 4318) | 762(136 to 2187) | 6461(803 to 18123) | 1008(100 to 2943) | 0.54(0.15 to 0.94) |
| Low-middle | 656(134 to 1794) | 579(108 to 1632) | 2486(268 to 7112) | 844(76 to 2464) | 0.59(0.18 to 0.99) |
| Low | 174（32 to 493) | 459(79 to 1344) | 577(69 to 1714) | 634(72 to 1982) | 0.75(0.23 to 1.28) |

AAPC=average annual percentage change; CI=confidence interval; SDI=sociodemographic index; UI=uncertainty interval;.DAYLS=Disability-Adjusted Life Years.

**Supplementary Table 3 Main risk factors for Age standardised Alzheimer’s disease and other dementias related death, and AAPC, among people aged ≥65 years , 1991-2021**

| Risk factors by SDI | Age standardised death rate (95% UI))  1991 | Age standardised death  Rate (95% UI))  2021 | AAPC (95% CI)) | P value |
| --- | --- | --- | --- | --- |
| High fasting plasma glucose |  |  |  |  |
| Global | 94（4 to 301) | 135（5 to 428) | 1.21( 1.08 to 1.33) | ＜0.001 |
| High SDI | 105（4 to 332） | 156（6 to 490) | 1.43(1.29 to 1.58) | ＜0.001 |
| High-middle SDI | 93（4 to 303) | 127（5 to 409) | 1.05(0.94 to 1.16) | ＜0.001 |
| Low SDI | 62（2 to 199) | 90（3 to 295) | 0.97(0.76 to 1.18) | ＜0.001 |
| Low-middle SDI | 64（2 to 203) | 104（4 to 333) | 1.31(1.14 to 1.48) | ＜0.001 |
| Middle SDI | 86（3 to 281) | 115（4 to 377) | 0.89( 0.76 to 1.02) | ＜0.001 |
| High body-mass index |  |  |  |  |
| Global | 53（-4 to 245) | 70（-10 to 296) | 1.32(1.20 to 1.43) | ＜0.001 |
| High SDI | 72（-8 to 321) | 84（-12 to 348) | 0.875278 0.682121 1.068806 | ＜0.001 |
| High-middle SDI | 59（-6 to 274) | 85（-13 to 358) | 1.37(1.23 to 1.51) | ＜0.001 |
| Low SDI | 4（-2 to 36) | 18（-0.30 to 100) | 4.91(4.34 to 5.49) | ＜0.001 |
| Low-middle SDI | 13（-0.04 to 71) | 38（-4. to 164) | 3.18(2.93 to 3.36) | ＜0.001 |
| Middle SDI | 18（-0.10 to 99) | 48（-5 to 209) | 3.89(3.61 to 4.16) | ＜0.001 |
| Tobacco: |  |  |  |  |
| Global | 31(7 to 83) | -1( -1.53 to -0.45) | -0.99(-1.53 to -0.454) | ＜0.001 |
| High SDI | 36(8 to 96) | 23（6 to 59) | -1.36(1.98 to 0.74) | ＜0.001 |
| High-middle SDI | 27(6 to 71) | 24（5 to 66 ) | -0.36( -1.00 to 0.28) | 0.27 |
| Low SDI | 14（3 to 42) | 12（3 to 35) | -0.88 (-1.13 to -0.62) | ＜0.001 |
| Low-middle SDI | 23（5 to 65) | 18（4 to 51) | -1.003( -1.30 to -0.71) | ＜0.001 |
| Middle SDI | 29（7 to 79) | 22（5 to 62) | -1.014( -1.50 to -0.53) | ＜0.001 |

AAPC=average annual percentage change; CI=confidence interval; SDI=sociodemographic index; UI=uncertainty interval.

**Supplementary Figure 1**


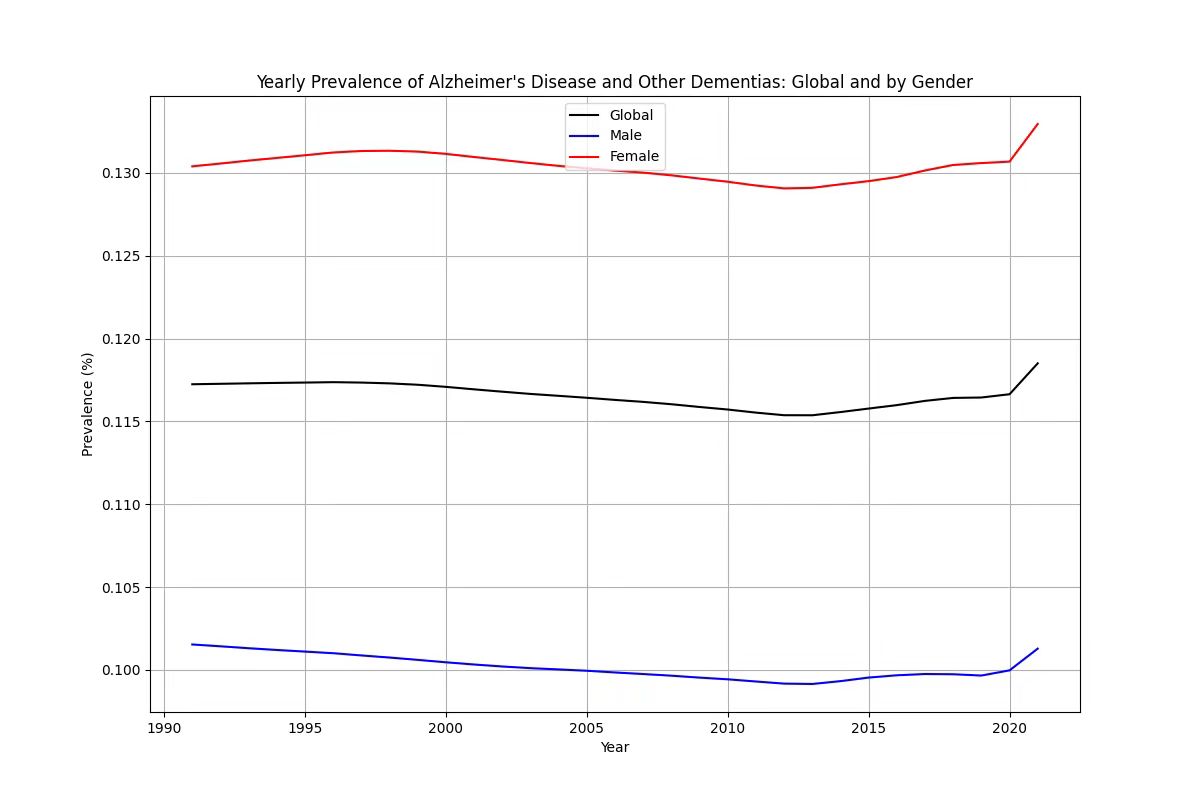


**Supplementary Figure 2**


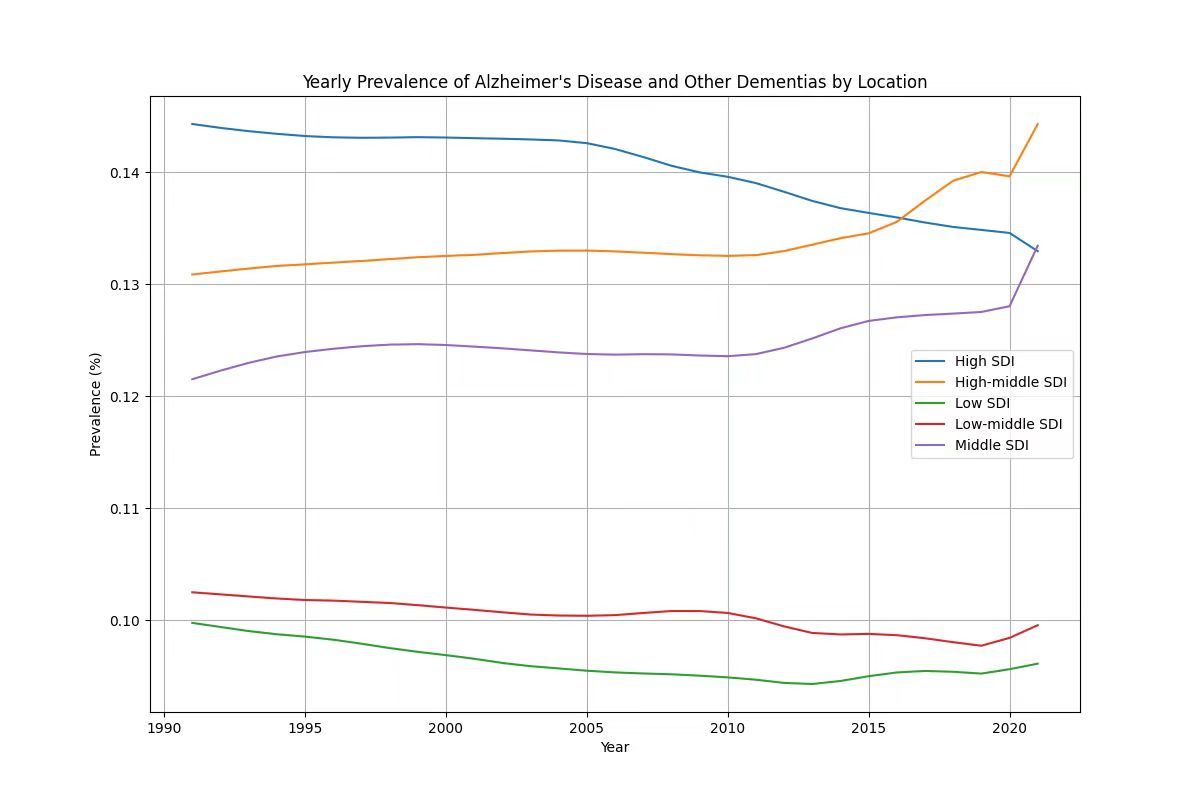


**Supplementary Figure 3**

**
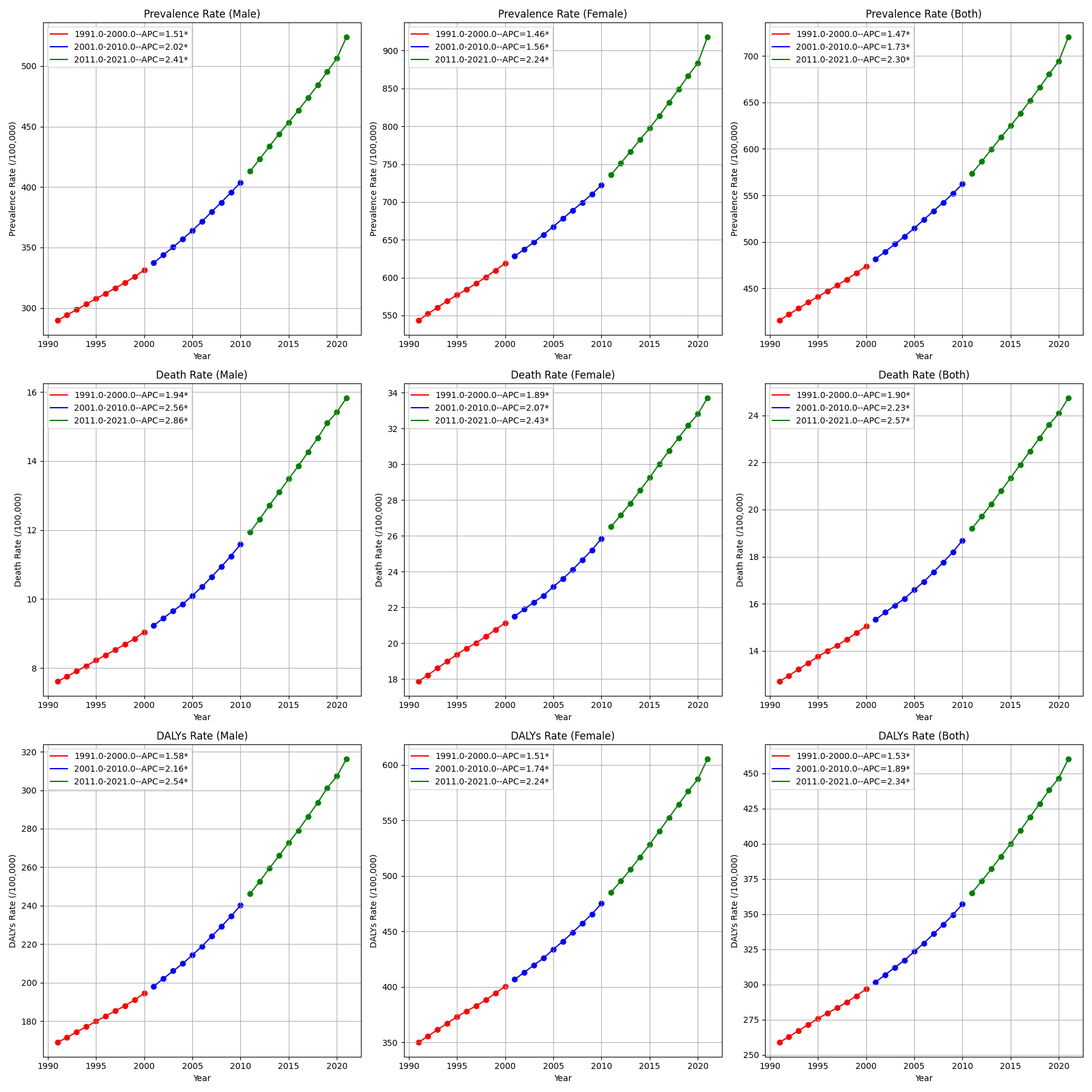
**

**Supplementary Figure 4**

**
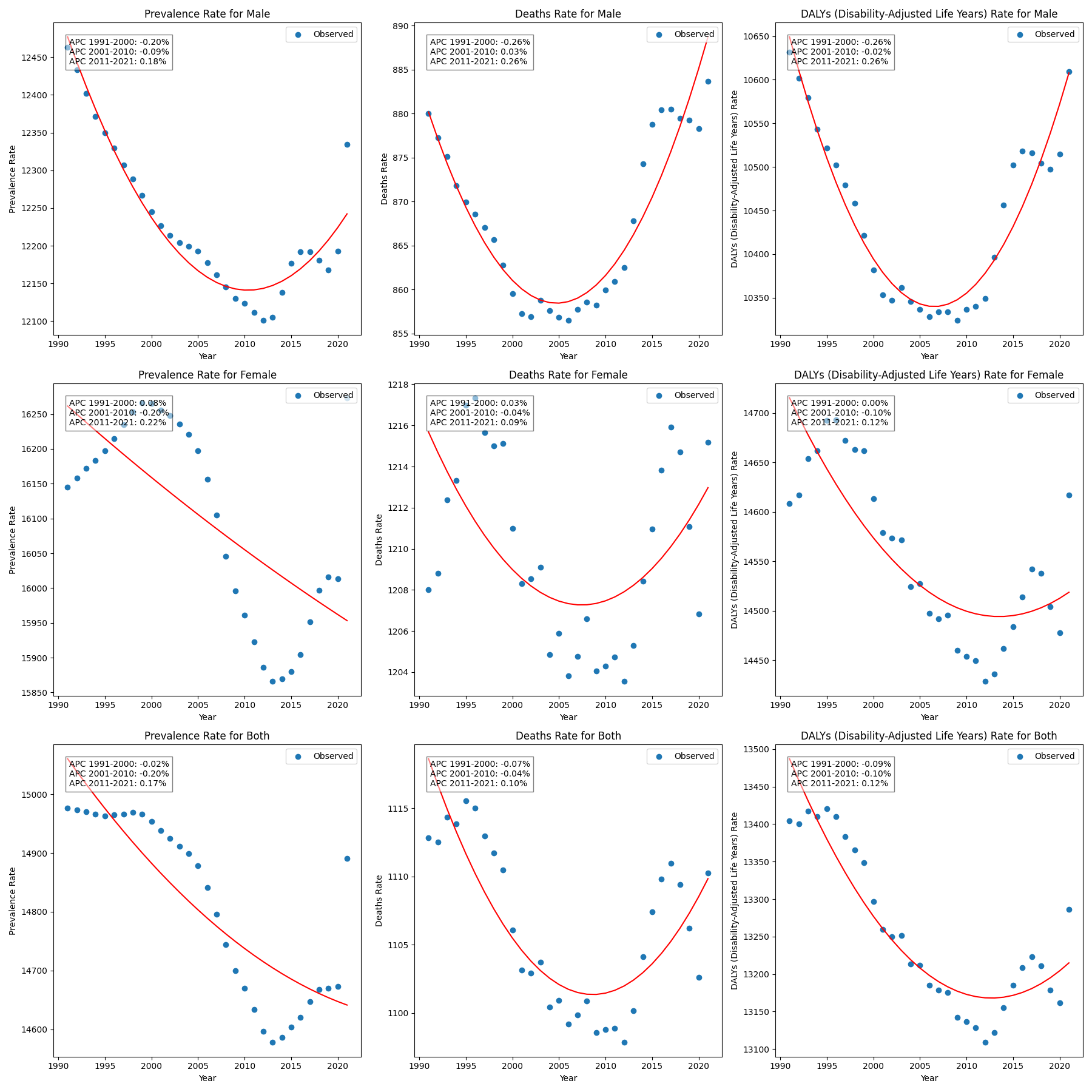
**

**Supplementary Figure 5**

**
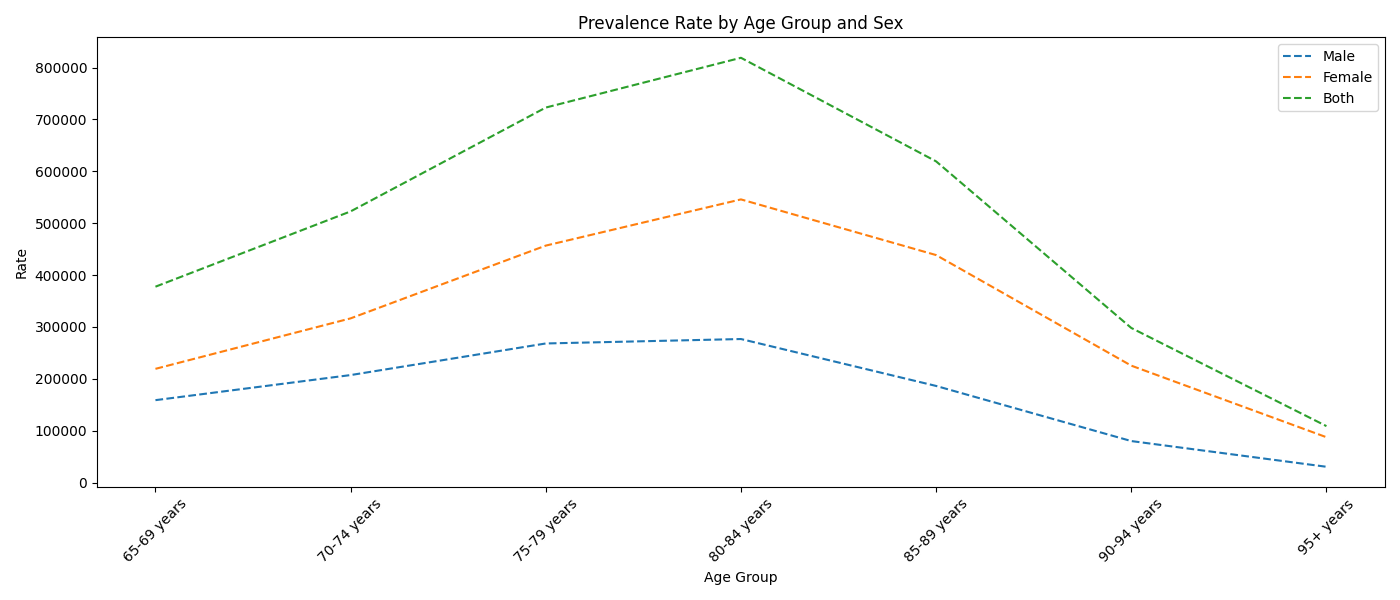
**

**Supplementary Figure 6**

**
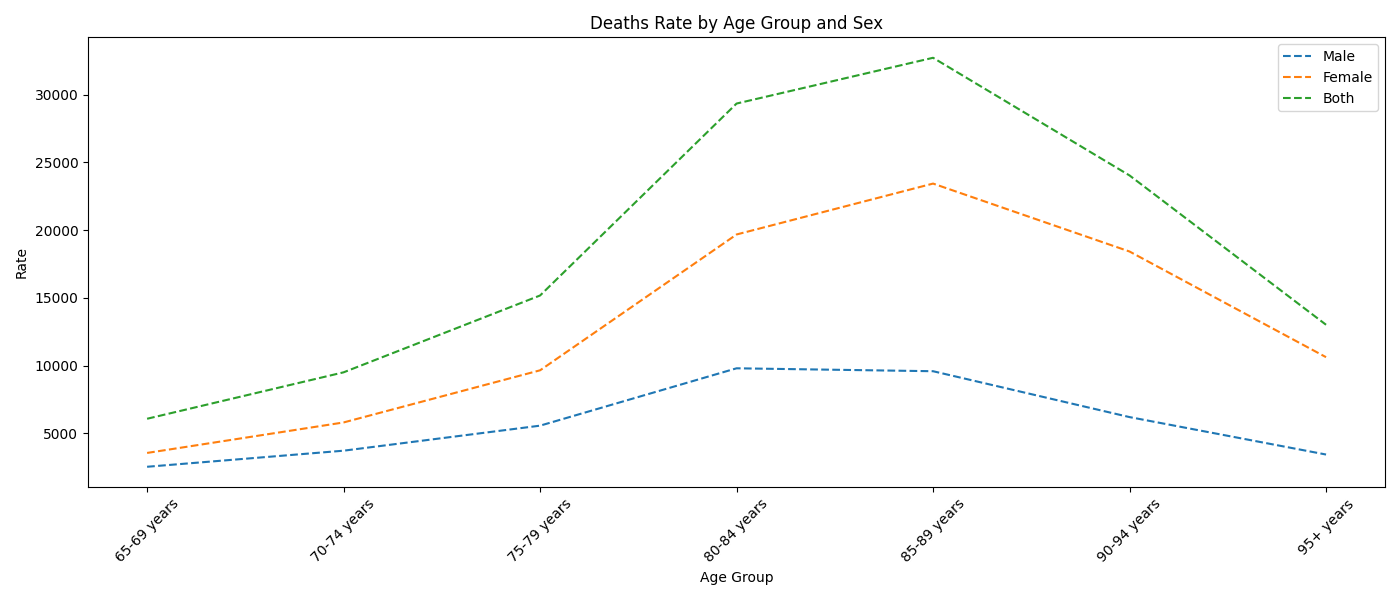
**

**Supplementary Figure 7**

**
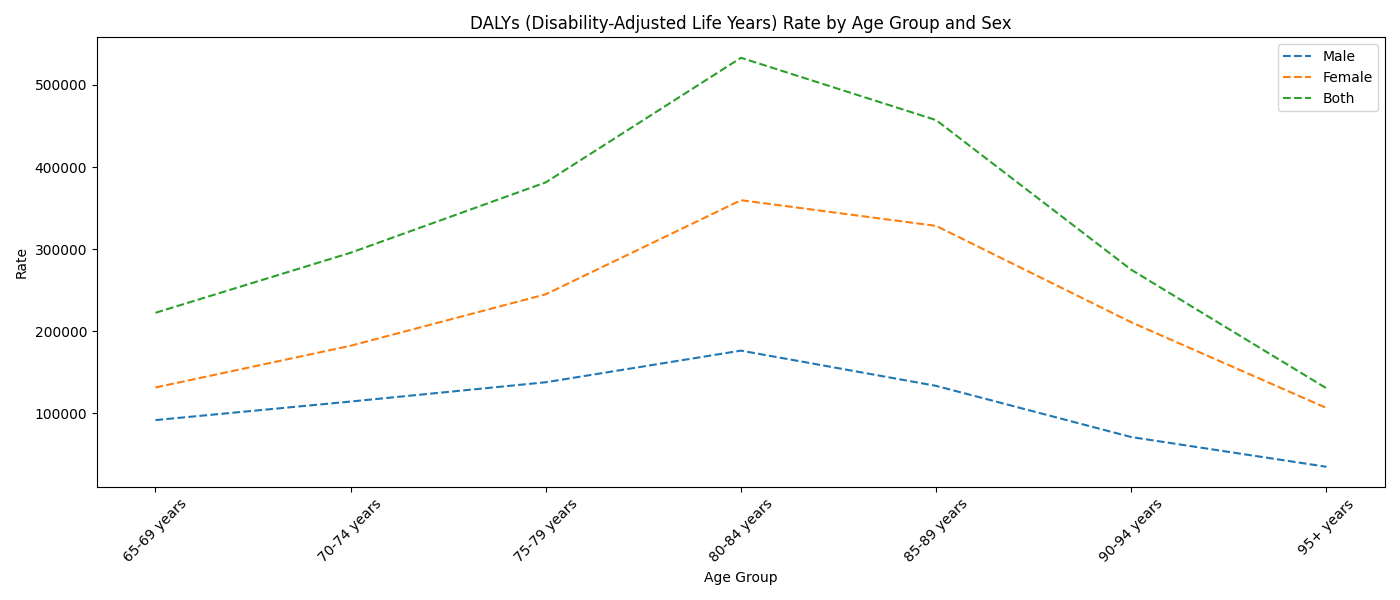
**

**Supplementary Figure 8**

**
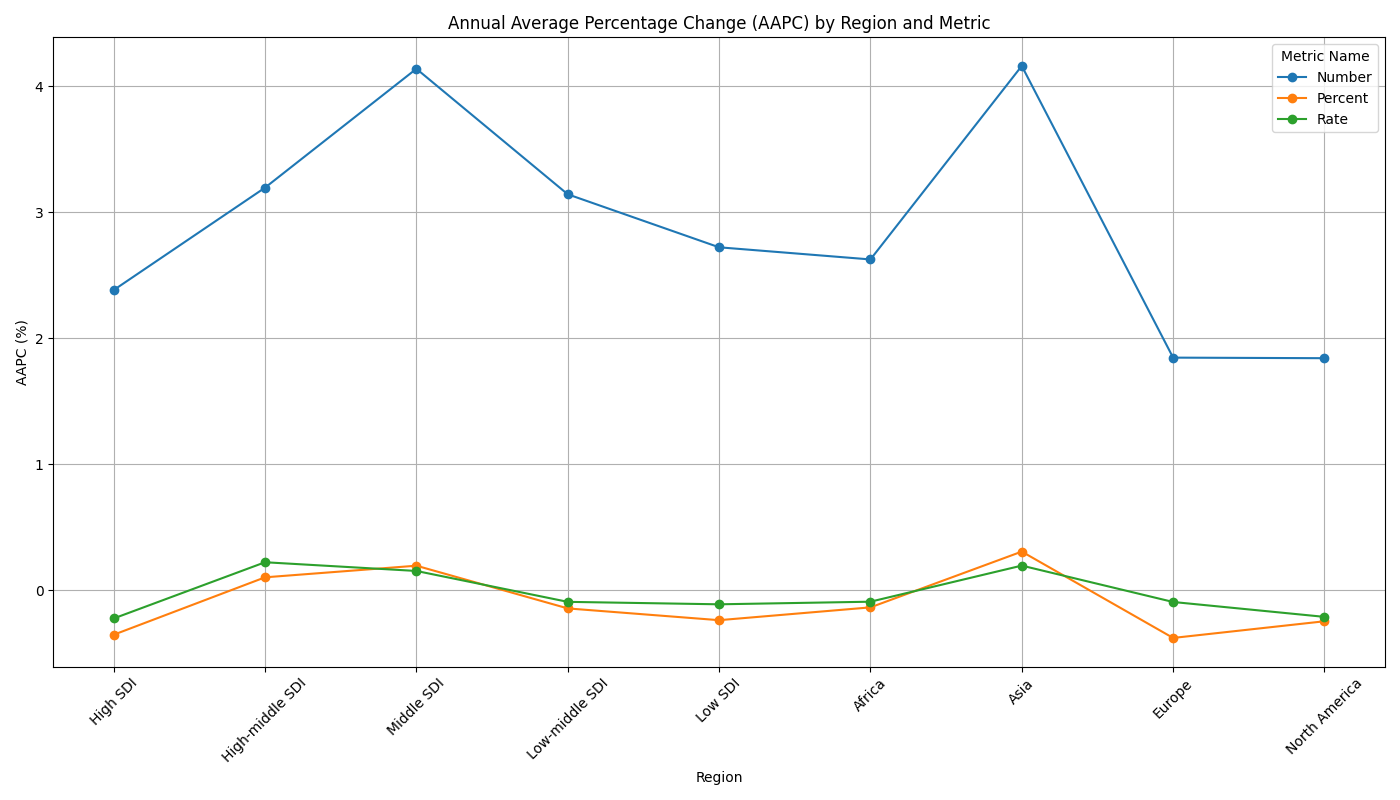
**

**Supplementary Figure 9**

**
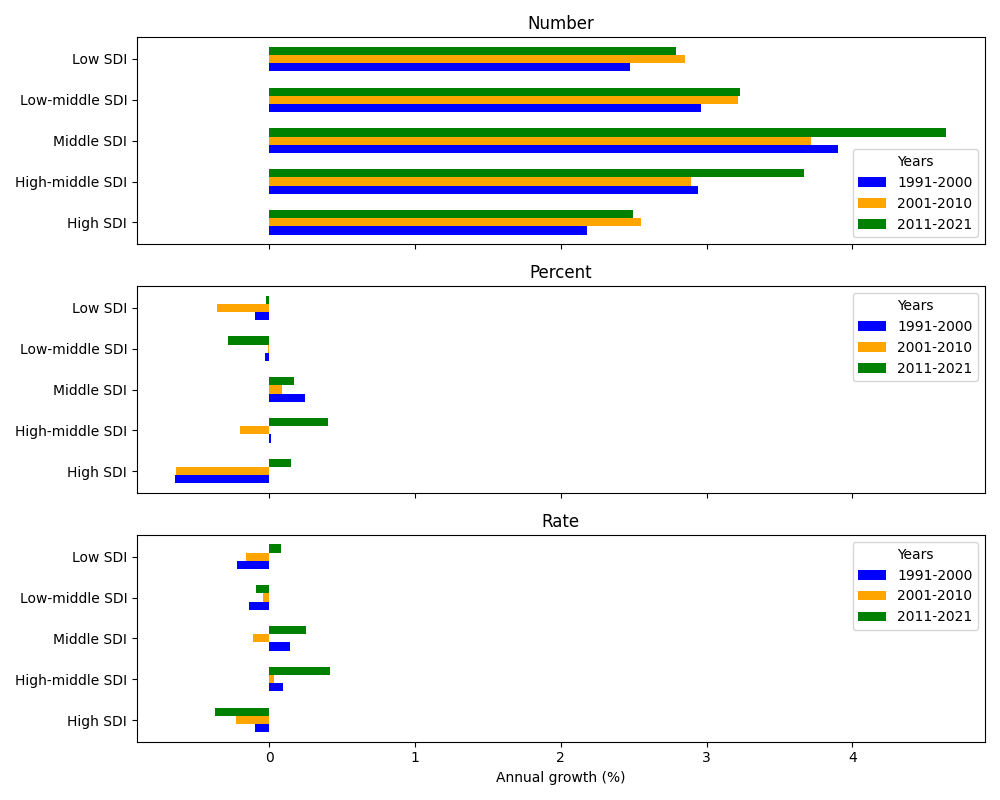
**

**Supplementary Figure 10**

**
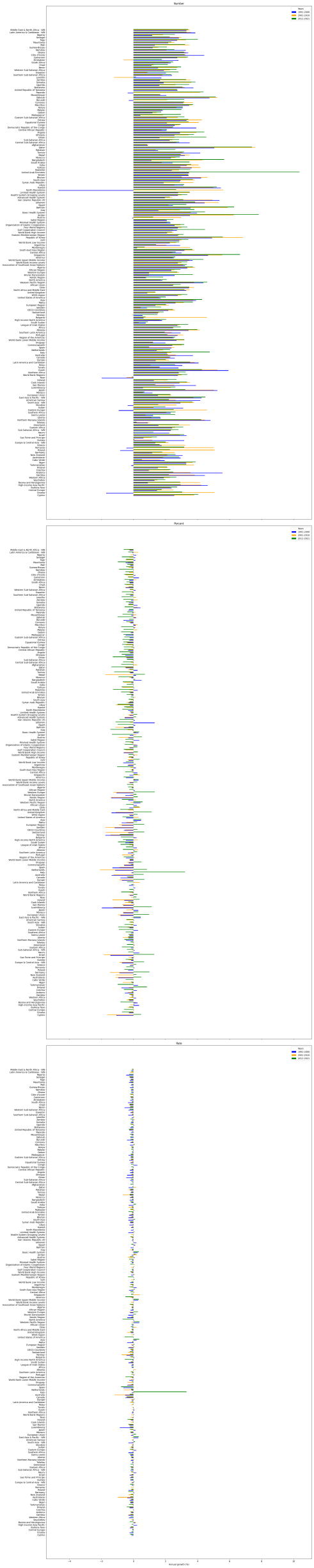
**
